# Gαq Protein Carboxyl Terminus Imitation Polypeptide GCIP-27 Improves Cardiac Function in Chronic Heart Failure Rats

**DOI:** 10.1371/journal.pone.0121007

**Published:** 2015-03-30

**Authors:** Xiao Lan Lu, Yang Fei Tong, Ya Liu, Ya Li Xu, Hua Yang, Guo Yuan Zhang, Xiao-Hui Li, Hai-Gang Zhang

**Affiliations:** 1 Department of Pharmacology, College of Pharmacy, Third Military Medical University, Chongqing 400038, China; 2 Department of Clinical Laboratory, First Affiliated Hospital of North Sichuan Medical College, Sichuan Nanchong 637000, China; 3 Institute of Materia Medica and Department of Pharmaceutics, College of Pharmacy, Third Military Medical University, Chongqing 40038, China; 4 Department of Ultrasound, Second Affiliated Hospital, Third Military Medical University, Chongqing 400037, China; Virginia Commonwealth University, UNITED STATES

## Abstract

**Background:**

Gαq protein carboxyl terminus imitation polypeptide (GCIP)-27 has been shown to alleviate pathological cardiomyocyte hypertrophy induced by various factors. Pathological cardiac hypertrophy increases the morbidity and mortality of cardiovascular diseases while it compensates for poor heart function. This study was designed to investigate the effects of GCIP-27 on heart function in rats with heart failure induced by doxorubicin.

**Methods and Results:**

Forty-eight rats were randomly divided into the following six groups receiving vehicle (control), doxorubicin (Dox), losartan (6 mg/kg, i.g.) and three doses of GCIP-27 (10, 30, 90 μg/kg; i.p., bid), respectively. Heart failure was induced by Dox, which was administered at a 20 mg/kg cumulative dose. After 10 weeks of treatment, we observed that GCIP-27 (30, 90 μg/kg) significantly increased ejection fraction, fraction shortening, stroke volume and sarcoplasmic reticulum Ca^2+^ ATPase activity of Dox-treated hearts. Additionally, GCIP-27 decreased myocardial injury, heart weight index and left ventricular weight index, fibrosis and serum cardiac troponin-I concentration in Dox-treated mice. Immunohistochemistry, western blotting and real-time PCR experiments indicated that GCIP-27 (10–90 μg/kg) could markedly upregulate the protein expression of myocardial α-myosin heavy chain (MHC), Bcl-2, protein kinase C (PKC) ε and phosphorylated extracellular signal-regulated kinase (p-ERK) 1/2 as well as the mRNA expression of α-MHC, but downregulated the expression of β-MHC, Bax and PKC βII, and the mRNA expression levels of β-MHC in Dox-treated mice. It was also found that GCIP-27 (30, 90 μg/L) decreased cell size and protein content of cardiomyocytes significantly *in vitro* by comparison of Dox group.

**Conclusions:**

GCIP-27 could effectively ameliorate heart failure development induced by Dox. PKC–ERK1/2 signaling might represent the underlying mechanism of the beneficial effects of GCIP-27.

## Introduction

Chronic heart failure (CHF) is a complex clinical syndrome resulting from any structural or functional cardiac disorder that impairs the systolic and/or diastolic ability of the ventricles. Among cardiovascular diseases, CHF is a leading cause of mortality and morbidity. Approximately 1%–2% of the adult population in developed countries suffers from CHF [1, 2]. Moreover, CHF is becoming more prevalent worldwide mainly because of the aging of the population and improved survival after acute cardiac events [3, 4]. A multitude of pharmacological approaches, including angiotensin converting enzyme inhibitors (ACEIs), angiotensin receptor blockers (ARB), and β receptor blockers have been widely used in clinical applications and have achieved remarkable outcomes during the past decade [5–8]. Despite this progress, the morbidity and mortality of CHF remain high; CHF is associated with an annual mortality rate of 10% [9]. Further exploration of new disease-modifying pharmacological targets remains one of the primary tasks in the prophylaxis and treatment of cardiac hypertrophy and CHF.

The pathophysiological changes in CHF are complicated [10, 11]. It has become clear recently that ventricular remodeling is the foundation of heart failure progression [10, 12]. It has been suggested that the discovery of molecular markers specific for different phenotypes of hypertrophic hearts could lead to effective treatments for specific cardiac hypertrophy [13]. Accordingly, the treatment of heart failure has changed direction and now focuses on preventing and even reversing ventricular remodeling [14].

Gαq protein carboxyl terminus imitation polypeptide (GCIP)-27 is a synthetic polypeptide that was previously designed and optimized in our laboratory [15]. Serial studies indicated that the GCIP-27 peptide could be preferentially transported into cardiomyocytes and vascular smooth muscle cells through an energy-dependent endocytosis mechanism [16], inhibiting cardiomyocyte hypertrophy induced by angiotensin II or norepinephrine in vitro and alleviating left ventricular hypertrophy in various animal models [15–19]. Based on these results, there is little doubt that GCIP-27 could ameliorate CHF by alleviating cardiac hypertrophy. However, cardiac hypertrophy is also regarded as an adaptive response to malignant stresses and is believed to have a compensatory function by diminishing wall stress and oxygen consumption as well as by maintaining cardiac output to the body [17, 20]. This finding has been demonstrated in a mouse model of pressure overload in which the inhibition of cardiac hypertrophy with cyclosporine A resulted in increased mortality caused by heart failure [13]. Whether GCIP-27 could impair heart function during the compensatory stage and heart failure stage by alleviating cardiac hypertrophy remains unclear. This study was designed to investigate the effects and mechanism of the GCIP-27 polypeptide drug on heart function in rats challenged with Dox that induces CHF.

## Materials and Methods

### Animals

Neonatal (1–2 days old) and ten-week-old male Sprague-Dawley rats were provided by the Experimental Animals Center of the Third Military Medical University (Chongqing, China). All animals were housed in an air-conditioned room with a 12-h light-dark cycle and fed standard chow and water *ad libitum*. The temperature and relative humidity were kept constant. All protocols conform to *The Guide to the Care and Use of Laboratory Animals* published by the Canadian Council on Animal Care (CCAC, http://www.ccac.ca/en/CCAC_Programs/Guidelines_Policies/GUIDES/ENGLISH/toc_v1.htm) and were approved by the Ethical Committee for Animal Experimentation of the Third Military Medical University.

### Treatments

**Fig 1:**
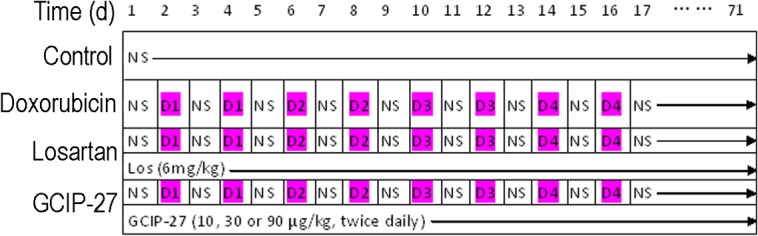
Drug administration schedule. NS, normal saline; Los, losartan (6 mg/kg, po., once daily); D1-D4, doxorubicin 1–4 mg/kg (ip., bid).

Animals were kept in the facility for one week to allow that animals to accustom to the new environment, forty-eight rats were randomly divided into the following six groups: control, doxorubicin-, losartan- and GCIP-27 (10, 30, 90 μg/kg)-treated groups. Doxorubicin (Dox, Wanle Pharmacy Co., Shenzhen, China) was dissolved in normal saline and administered intraperitoneally at a dose of 1 mg/kg on the 2^nd^ and 4^th^ days, 2 mg/kg on the 6^th^ and 8^th^ days, 3 mg/kg on the 10^th^ and 12^th^ days, and 4 mg/kg on the 14^th^ and 16^th^ days [21]. An accumulated dose of 20 mg/kg Dox was administered to all of the animals, except those in the control group, at the scheduled time (Fig. 1). The rats in the GCIP-27-treated groups were injected intraperitoneally with 10, 30 or 90 μg/kg of GCIP-27 (purity>98%, synthesized by Scilight Biotechnology Co., Beijing, China) dissolved in normal saline (3 ml/kg) twice daily. The rats in the control group were injected with the identical volume of normal saline, and the rats in the losartan-treated group were given 6 mg/kg of losartan (Los, purity>98%, Kerui Pharmaceutical Co., Ltd., Chongqing, China) intragastrically once daily.

Humane end points were set according to the OECD *Guidance Document on the Recognition*, *Assessment*, *and Use of Clinical Signs as Humane End points for Experimental Animals Used in Safety Evaluation* (https://www.aaalac.org/accreditation/RefResources/RR_HumaneEndpoints.pdf). Specifically, as one was found showing body temperature drop, appearance of hunched and starey coat, or decreased activity with no response to touch, the rat was sacrificed by overdose of pentobarbital. All of the animals survived were treated for 10 weeks and were weighed weekly, and the doses were adjusted accordingly.

### Cell culture and treatment

Neonatal rats ventricular myocytes (NRVC) from 1–2 days old Sprague-Dawley rats were isolated and cultured as described previously [15, 16]. NRVC were washed twice with D-Hanks' solution after being cultured in serum-free DMEM for 24 h, and incubated for 6 h in a non-serum medium containing 0.1 μmol/L DOX. Different concentrations of GCIP-27 (3, 10 and 30 μg/L) or losartan (10 μmol/L) were added to observe its effects. The cell size was determined with fluorescent phalloidin staining and analyzed with ImageJ software (NIH Image, National Institutes of Health, Bethesda, MD; online at: http://rsbweb.nih.gov/ij/) [22]. And the protein content of cardiomyocytes were measured with the method of Lowry et al using bovine serum albumin as the standard [15] according to the manufacturer's guide.

### Echocardiography

After 10 weeks of treatment (described above), all of the rats that survived were subjected to echocardiography measurements [17, 23] under anesthesia with chloral hydrate (350 mg/kg, ip). Transthoracic two-dimensional and M-mode images were performed in animals using a Vivid 7 Echo machine with the 14 MHz probe (GE Medical Systems, Minneapolis, MN). M-mode measurements of the left ventricular internal end-diastolic diameter (diastolic LVID) and the posterior wall end-diastolic thickness (diastolic LVPW) were determined as suggested by the American Society of Echocardiography. Simultaneously, the LV diastolic volume (diastolic LVV) and function indexes (stroke volume, ejection fraction and fraction shortening) were calculated according to the results of the echocardiography [24]. An experienced sonographer blinded to the treatment groups performed all of the studies. Two observers, blinded to the treatment assignments, analyzed the images.

### Tissue Sampling

After recording the echocardiogram, the rats were sacrificed under anesthesia for sampling. The hearts were removed rapidly and washed with normal saline, dried with filter paper and weighed. The atria and free wall of right ventricles were removed, and the interventricular septum was remained. The heart weight index and LV weight index were calculated as the ratio of the heart weight (HW) and left ventricle weight (LVW) to body weight (BW) or tibial length (TL), respectively [15, 17, 25]. The mid ventricle was fixed with a formalin neutral buffer solution and embedded in paraffin. The apex of the ventricle was cryopreserved with liquid nitrogen for future use.

### Histopathological Analysis

Paraffin-embedded sections of the mid ventricle were stained with hematoxylin and eosin (H&E), as well as Masson’s trichrome [26]. Cross-sectional area (CSA) in each group was acquired with a microscopic digital camera system, Nikon ECLIPSE E100 (Nikon, Tokyo, Japan). The fibrosis level was evaluated with a 0–10 scale [27].

### Plasma troponin I assay

The plasma cardiac troponin I (cTnI) levels were determined quantitatively using the immunochemiluminescence method (Architect i2000sr; Abbot Diagnostics, IL, USA) with an assay kit (Abbot Diagnostics) according to the manufacturer’s protocol.

### Sarcoplasmic reticulum Ca^2+^ ATPase Measurements

To investigate the effects of the GCIP-27 treatment on the myocardial sarcoplasmic reticulum Ca^2+^ ATPase (SERCA2a) activity, proteins were extracted and quantified from heart tissue samples taken from the left ventricular region, as previously described. SERCA2a activity was measured using a Ca^2+^-ATPase assay kit (Sunbio Biotech, Beijing, China) according to the manufacturer’s instructions. SERCA2a activity was normalized to the protein concentration [28].

### Immunohistochemistry

The myocardial expression levels of α- and β-myosin heavy chain (MHC), Bax, as well as Bcl-2 were detected using immunohistochemistry techniques. Sections (10 μm) from paraffin-embedded tissues were stained using the simplified histostain SP-kit (Zymed Laboratories, USA) according to the manufacturer's instructions. After deparaffinization and rehydration and the inhibition of endogenous peroxidase, the sections were exposed to primary antibodies at 4°C overnight. After incubation with a secondary antibody at room temperature, the sections were incubated in 3,3 N-diaminobenzidine tetrahydrochloride (DAB) and counterstained with hematoxylin. The following primary antibodies were used at a dilution of 1:150: anti-α-MHC, anti-β-MHC, anti-Bax and anti-Bcl-2 (Santa Cruz Biotechnology, CA, USA). The secondary antibody was goat-anti-mouse immunoglobulin conjugated with horseradish peroxidase (dilution of 1:200). Digital images of the stained sections were acquired using a Nikon ECLIPSE E100 microscope with a digital camera system. Unanimity regarding positive immunohistochemical staining in each preparation was reached by two blinded investigators using Image-Pro Plus 5.1 software (Media Cybernetics, Silver Spring, MD).

### RNA preparation and real-time reverse transcription-PCR

Total RNA was isolated from the ventricular tissue using the Tripure reagent (Invitrogen, USA) according to the manufacturer’s instructions. The RNA samples were dissolved in nuclease-free water and treated with 5 U of DNase I (Takara, Shiga, Japan) for 30 min at 37°C. The reaction was stopped by the addition of 25 mmol/L EDTA and a 15-min incubation at 65°C. The total RNA concentration was quantified by measuring the absorbance at 260 nm. Total RNA (1 μg) was reverse transcribed using AMV reverse transcriptase (TOYOBO, Osaka, Japan) at 42°C for 1 h. The PCR primers used were designed by Premier 5.0 (PREMIER Biosoft International, Palo Alto, CA, USA) based on the published nucleotide sequences for rat α-MHC (forward: 5'-TAT GCT GGC ACC GTG GAC TA-3'; reverse: 5'-GAG TTT GAG GGA GGA CTT CTG G-3'), β-MHC (forward: 5'-GGG CAA AGG CAA AGC AAA GA-3’; reverse: 5'-AAA GTG AGG GTG CGT GGA GC-3'), and β-actin (forward: 5'-CGT AAA GAC CTC TAT GCC AAC A-3’; reverse: 5'-TAG GAG CCA GGG CAG TAA TC-3'). Each real-time PCR reaction was performed in triplicate in a total volume of 25 μl with QPK-201 SYBR Green PCR Master Mix (TOYOBO, Osaka, Japan) using the following conditions: 5 min at 94°C, 38 cycles at 94°C for 30 s, annealing at 58°C for 30 s, 72°C for 45 s, and 82.5°C for 5 s (collecting fluorescence) with the ABI Prism 7700 sequence detection system (ABI, Oyster Bay, NY, USA). After amplification, a melting curve analysis was performed by collecting fluorescence data while increasing the temperature from 72°C to 99°C over 135 s. The Ct (cycle threshold) values were normalized to the β-actin expression levels.

### Western blot assay

The myocardial expression levels of protein kinase (PK)-Cε, -βII and phosphor-extracellular signal regulated kinase (pERK)1/2 were assayed by Western blot. Total cellular homogenates were prepared, and equal amounts (20 μg) of the denatured proteins were loaded and separated using sodium dodecyl sulfate polyacrylamide gel electrophoresis. The proteins were transferred onto a polyvinylidene difluoride membrane (Millipore, Bedford, MA) and incubated with a primary antibody (PKCε, PKCβII and p-ERK1/2 (Santa Cruz Biotechnology, Inc., USA)) at 4°C overnight, followed by horseradish peroxidase (HRP)-conjugated anti-goat IgG or HRP-conjugated anti-mouse IgG. Chemiluminescence was detected with an ECL western blot detection kit (Millipore, Bedford, MA) according to the supplier’s recommendations. The data were analyzed by an observer who was blind to the treatment given to the rats.

### Statistical analysis

All values are expressed as the mean ± SEM. The differences between the groups were assessed by one-way ANOVA with least significant difference (LSD) post-hoc analyses. The differences were considered significant when P<0.05. All of the statistical analyses were performed using the Statistical Package for Social Sciences for Windows software, version 10.0J (SPSS Co., Inc., Chicago, IL).

## Results

### General condition and mortality

The animals were observed continuously for 10 weeks. All of the rats in the Dox group showed weight loss, lethargy and less activity. In contrast to those treated with Dox, the rats in the control group, losartan group, and GCIP-27 groups were more healthy and active. During the entire experimental period, one rat in control group, 3 rats in Dox, Los and 10 μg/kg GCIP-27 group, 2 rats in 30 and 90 μg/kg GCIP-27 group, respectively, were sacrificed before the humane endpoints. There were no significant differences in the cumulative mortality between all the groups.

### Echocardiography Measurements

**Fig 2:**
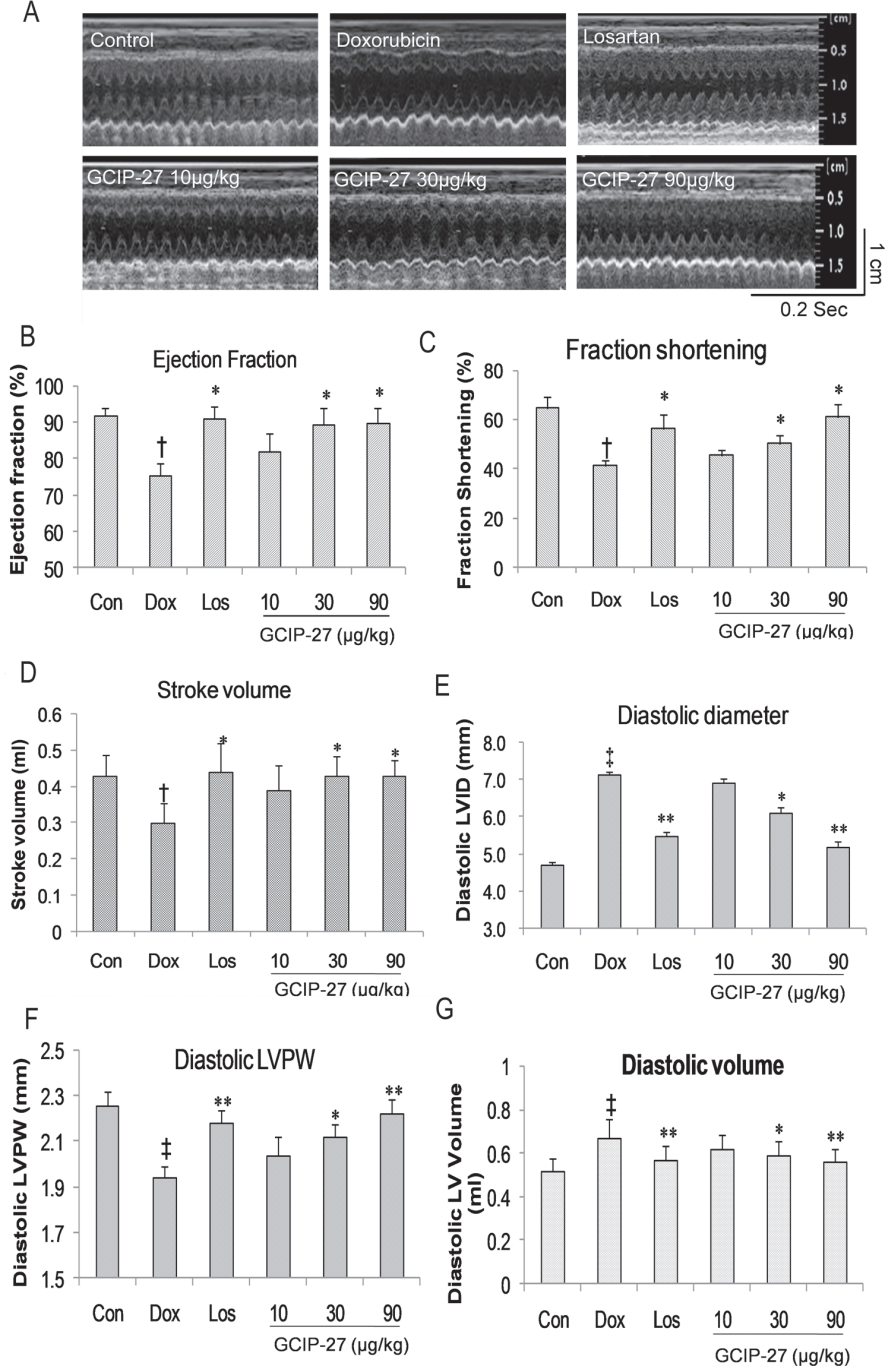
Cardiac function and structure were measured with echocardiography. (A). Ejection fraction (B), fraction shortening (C), stroke volume (D), left ventricular internal diastolic diameter (E), left ventricular posterior wall (LVPW) end diastolic thickness (F), and left ventricular end diastolic volume (G) were calculated with the formula stored in the echo machine by the manufacturer. The data are presented as the mean ± SEM (n = 5–7 in each group). Scale for time = 0.2 sec; scale for length = 1cm. ^†^ *P* < 0.05, ^‡^ *P* < 0.01 compared with the control group (Con); *P < 0.05, **P < 0.01 compared with the Dox group (one-way ANOVA).

The structure and function of the left ventricles were measured using high frequency echocardiography (Fig. 2A). The diastolic LVID and LVV in the Dox group increased significantly compared with that in the control group (Fig. 2E and G; *P* < 0.01). The diastolic LVPW, left ventricular ejection fraction (EF), fraction shortening (FS) and stroke volume in the Dox group were markedly decreased compared with those in the control group (Fig. 2B, C, D, F; *P* < 0.05 or 0.01). Doses of 30 and 90 μg/kg of GCIP-27 and losartan, but not 10 μg/kg of GCIP-27, ameliorated the aforementioned indexes compared with those in the Dox group (Fig. 2B-G, *P* < 0.05 or 0.01).

### Heart indexes

**Fig 3:**
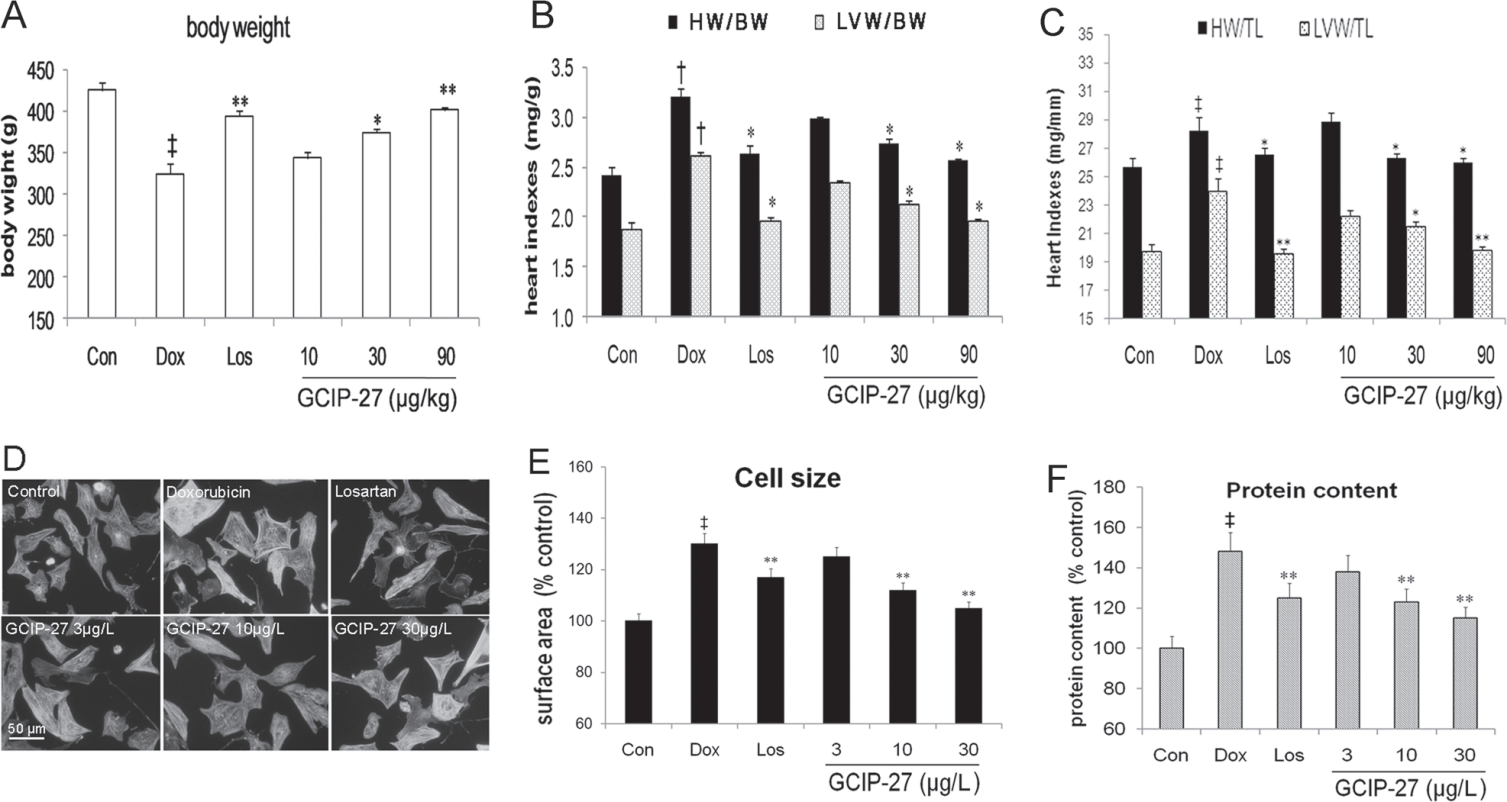
The effects of the GCIP-27 on hypertrophic response in cardiomyocytes *in vitro* and heart indexes in rats with heart failure induced by doxorubicin. Chronic heart failure was induced by a cumulative dose of 20 mg/kg doxorubicin (Dox). (A) body weight (n = 5–7 in each group); (B) Heart and left ventricular (LV) weight index to body weight; (C) Heart and LV indexes to tibia length; (D) Neonatal rats ventricular cells treated with Dox (0.1 μmol/L) and Los or GCIP-27 for 6 h, stained with FITC-phalloidin (bar = 50 μm); (E) cell size (n = 30 cells in each group); (F) protein content (n = 6 wells in each group)The data are presented as the mean ± SEM. ^‡^ *P* < 0.01 compared with the control group (Con); *P < 0.05, **P < 0.01 compared with the Dox group (one-way ANOVA).

The heart index HW/BW and HW/TL, LV index LVW/BW and LVW/TL of the rats were measured to explore cardiac hypertrophy. Although Dox markedly decreased the body weight compared with that in the control group, the treatment with GCIP-27 (30 and 90 μg/kg) and losartan obviously ameliorated weight loss (*P* < 0.01 or 0.05, Fig. 3A). Additionally, Dox increased heart index and LV index significantly (*P* < 0.05, Fig. 3B and 3C). GCIP-27 (30 and 90 μg/kg) and losartan remarkably decreased heart index and LV index compared with those of the rats in the Dox group (*P* < 0.01). There was no significant difference between the GCIP 10 μg/kg group and the Dox group.

### Cell size and protein content

Surface area and protein content of primary cardiomyocytes were measured to assess its hypertrophic response induced by Dox. As shown in Fig. 3D-F, at a dose of 0.1 μmol/L, Dox increased cell size and protein content significantly (*P* < 0.01), and treatment of Los or GCIP-27 (10 and 30 μg/L) reduced cardiomyocytes size and protein content (*P* < 0.01), while low dose of GCIP-27 (3 μg/L) had no significant influence on them (*P >0*.*05*).

### Pathological Findings

**Fig 4:**
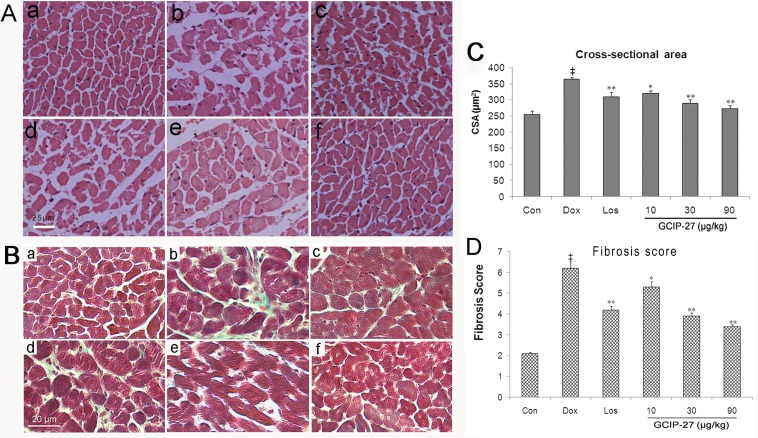
Myocardial pathological changes after treatment with GCIP-27. Chronic heart failure was induced by a cumulative dose of 20 mg/kg doxorubicin (Dox). (A) H&E staining (bar = 25 μm): (a) control, (b) Dox, (c) losartan (Los) 6mg/kg, (d) GCIP-27 10 μg/kg, (e) GCIP-27 30 μg/kg, (f) GCIP-27 90 μg/kg; ip., bid; (B) Cross-sectional area; (C) Masson’s Trichrome staining (bar = 20 μm), panel (a)-(f) represent same group as that in (A); (D) Fibrosis score. The data are presented as the mean ± SEM (n = 5–7 in each group). ^‡^ *P* < 0.01 compared with the control group (Con); *P < 0.05, **P < 0.01 compared with the Dox group (one-way ANOVA).

The myofibrils were lined up without disruption, and the structure of the nuclei and cells were normal in the control group. In the Dox group, myocardial fiber disruption and disarray could be observed, and the hyperplastic cardiomyocytes were infiltrated with inflammatory cells. Additionally, CSA and fibrosis score increased significantly (*P* < 0.01) compared with control group (Fig. 4). After treatment with 30 or 90 μg/kg of GCIP-27, the injury and pathological changes of the myocardium tissue improved markedly with less inflammatory cell infiltration and fiber disarray. After these treatments, CSA and fibrosis score decreased significantly (Fig. 4C and 4D).

### Plasma cTnI concentration and cardiac SERCA2a activity

**Fig 5:**
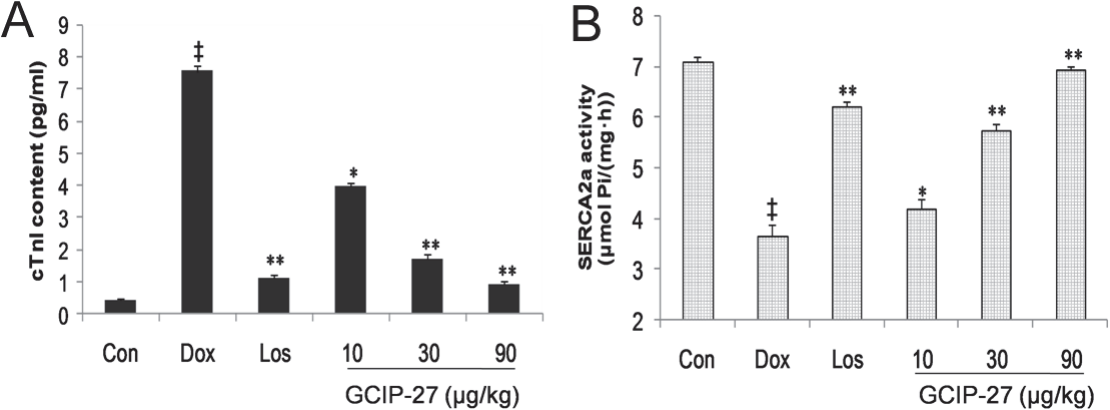
The effects of the GCIP-27 on serum cardiac troponin-I (cTnI) (A) and myocardial sarcoplasmic reticulum Ca^2+^ ATPase (SERCA2a) activity (B) in rats with doxorubicin-induced heart failure. Chronic heart failure was induced by a cumulative dose of 20 mg/kg doxorubicin (Dox). The rats were treated with normal saline, losartan (Los, 6 mg/kg, ig, once daily) or GCIP-27 (10, 30, 90 μg/kg, ip, bid) for 10 weeks. The data are presented as the mean ± SEM (n = 5–7 in each group). ^‡^ *P* < 0.01 compared with the control group (Con); *P < 0.05, **P < 0.01 compared with the Dox group (one-way ANOVA).

To evaluate the biochemical foundation of the systolic and diastolic function of the heart, plasma cTnI and ventricular SERCA2a activity were determined. The Dox treatment induced a marked increase in cTnI content compared with the control group (*P* < 0.01) (Fig. 5A). Simultaneously, the GCIP-27 (10, 30, 90 μg/kg) and losartan treatments decreased the cTnI concentration significantly compared with that of the Dox group (*P* < 0.05 or 0.01). In sharp contrast to the increase of plasma cTnI, Dox obviously reduced the SERCA2a activity, and the GCIP-27 treatment elevated it significantly (Fig. 5B). The effects of GCIP-27 on the cTnI content and SERCA2a activity were obviously dose-dependent.

### Myocardial expressions of α-MHC and β-MHC

**Fig 6:**
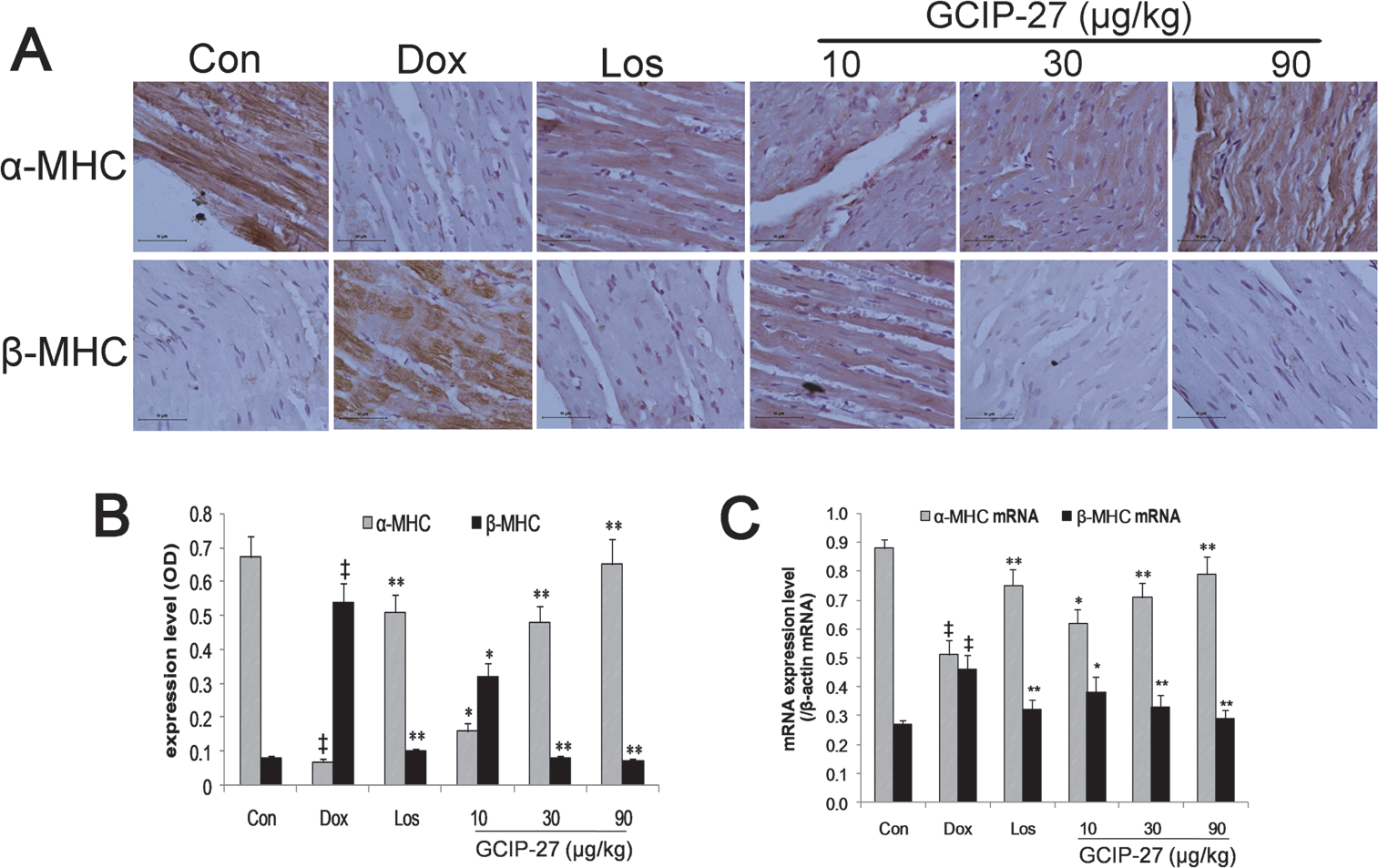
The effects of GCIP-27 on the myocardial expression of myosin heavy chains (MHCs). (A) The expression levels of α-MHC and β-MHC were determined with immunohistochemistry (bar = 10 μm). (B) Myocardial expression (mean ± SEM) of α- and β-MHC. (C) The myocardial mRNA expression of α- and β-MHC measured with real-time PCR. The data are presented as the mean ± SEM (n = 5–7 in each group). ^‡^ *P* < 0.01 compared with the control group (Con); *P < 0.05, **P < 0.01 compared with the Dox group (one-way ANOVA).

The baseline myocardial expression of α-MHC and β-MHC were shown as controls in Fig. 6A. The expression level of α-MHC in the Dox group was markedly decreased compared with that of the control group. The GCIP-27 (10, 30 and 90 μg/kg) and losartan treatments significantly elevated the α-MHC levels compared with those of the Dox group (Fig. 6A and 6B; *P* < 0.01). The expression of β-MHC showed an opposite trend compared with that of α-MHC. The ratio of the expression of α-MHC to that of β-MHC had a similar change pattern as that shown for α-MHC; this change pattern was more significant. Treatment with GCIP-27 (10, 30 and 90 μg/kg) and losartan noticeably elevated the myocardial expression of α-MHC mRNA compared with that of the Dox group, whereas they decreased the expression of β-MHC mRNA (Fig. 6C; *P* < 0.01 or 0.05).

### Expression of Bax and Bcl-2

**Fig 7:**
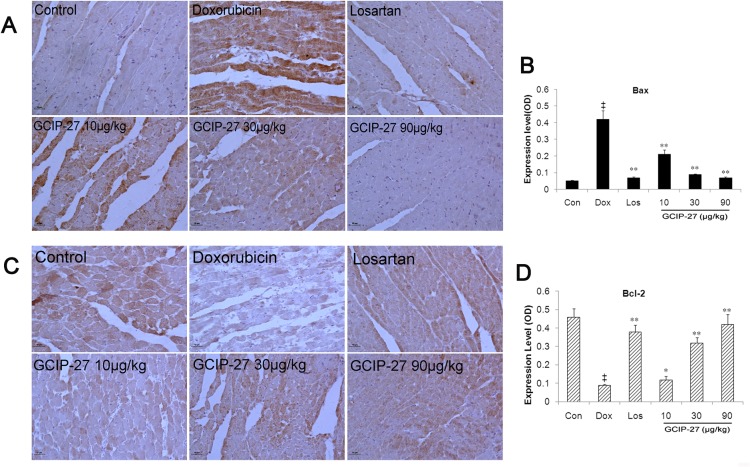
The effects of GCIP-27 on the myocardial expression of Bax and Bcl-2. The expression levels of Bax (A) and Bcl-2 (C) were determined with immunohistochemistry (bar = 10 μm). Bar graphs representing the myocardial expression of Bax (B) and Bcl-2. (D). The data are presented as the mean ± SEM (n = 5–7 in each group). ^‡^ *P* < 0.01 compared with the control group (Con); *P < 0.05, **P < 0.01 compared with the Dox group (one-way ANOVA).

Myocardial expression of Bax and Bcl-2 were measured to evaluate the effect of GCIP-27 on apoptosis induced by Dox. As shown in Fig. 7, the expression of Bax increased and that of Bcl-2 reduced significantly in Dox group compared with controls. Treatment of Los and GCIP-27 (10–90 μg/kg) reduced myocardial Bax expression and elevated Bcl-2 expression significantly (*P* < 0.01 or 0.05).

### Expression of PKCβII, PKC and p-ERK1/2

**Fig 8:**
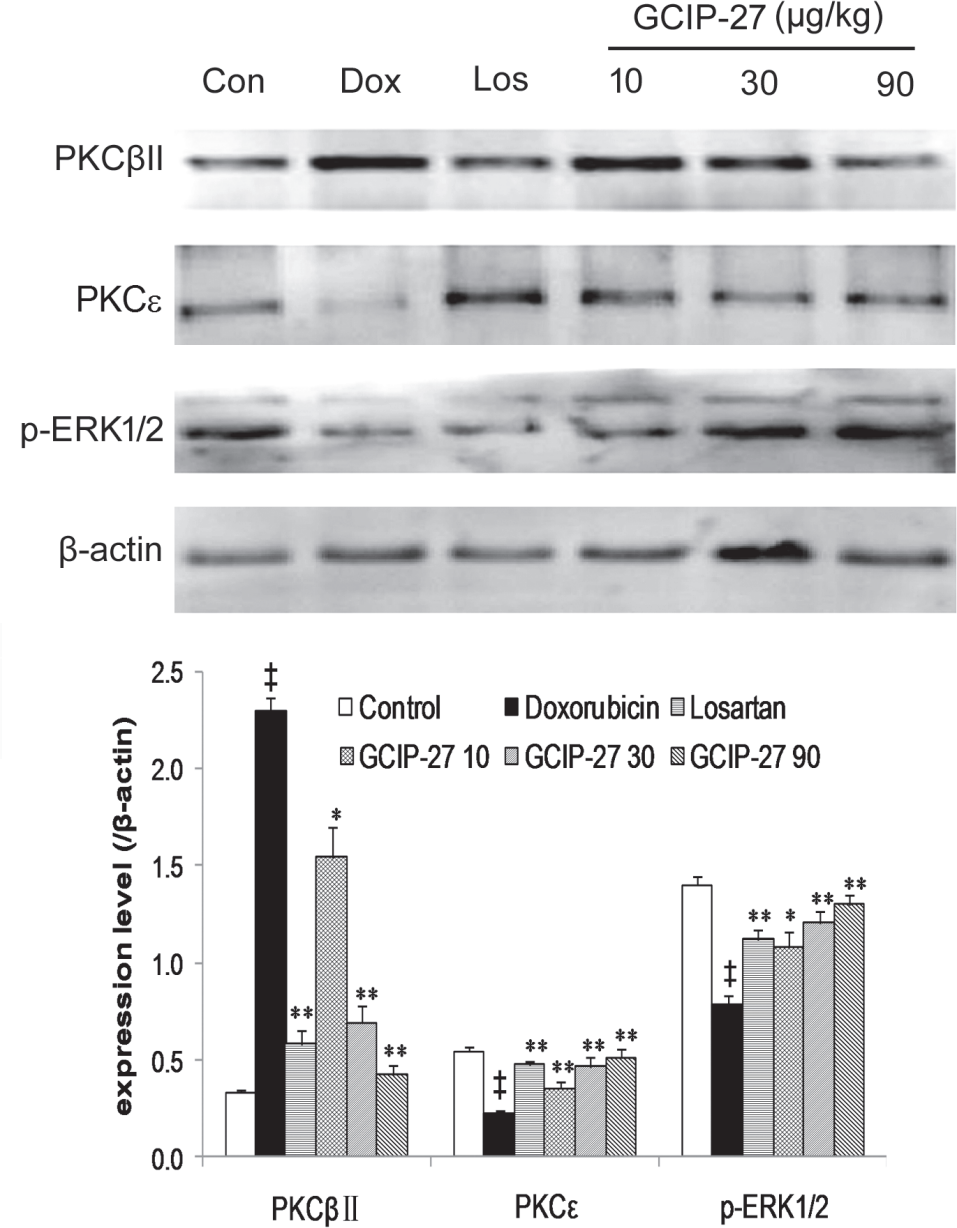
The expression of protein kinase C (PKC) βII, PKCε and p-extracellular signal-regulated kinase (ERK). (A) Western blot; (B) Averaged myocardial expression (mean ± the SEM) of the proteins mentioned above. ^†^ *P* < 0.05, ^‡^ *P* < 0.01 compared with the control group; *P < 0.05, **P < 0.01 compared with the doxorubicin group (one-way ANOVA).

Myocardial expression of PKCβII, PKC and p-ERK1/2 was detected by Western blotting (Fig. 8A). Compared with the control group, the expression of PKCβⅡincreased while the PKC expression decreased significantly in the Dox group (Fig. 8B; *P* < 0.01). Treatment with GCIP-27 (10, 30 and 90 μg/kg) and losartan effectively ameliorated these abnormal expressions compared with those in the rats in the Dox group (*P* < 0.01 or 0.05). Simultaneously, Dox reduced the myocardial expression of p-ERK1/2 (*P* < 0.01), and GCIP-27 (10, 30 and 90 μg/kg) elevated its expression compared with the Dox group (*P* < 0.05 or 0.01).

## Discussion

Several studies have demonstrated a beneficial effect of the GCIP-27 polypeptide on cardiac hypertrophy induced by a variety of factors in vivo and in vitro [15–19]. Cardiac hypertrophy is an important factor in the development of CHF. Cardiac hypertrophy is typically believed to be a compensatory mechanism of the heart in response to increased systemic demand for cardiac output. Given this premise, preventing or reversing ventricular remodeling might impair heart function during the compensatory stage and the heart failure stage. As GCIP-27 may reduce cardiac hypertrophy, it becomes important to determine whether GCIP-27 is beneficial to heart functioning during CHF. In this study, GCIP-27 treatment was shown to markedly increase body weight, improve survival, and halt the process of hypertrophy and heart failure in doxorubicin-induced CHF rats, preventing adverse ventricular remodeling imposed by Dox.

Due to the cardiac toxicity and other adverse effects of doxorubicin, rats receiving Dox may eat less and gain less body weight [29]. Along with the decrease of the heart function, concentration of plasmic catecholamine, such as norepinephrine (NE) increase significantly [30], which can reduce appetite and food intake through α_1_-adrenoceptor [31]. GCIP-27 is a synthetic peptide, which imitate the structure of carboxyl terminus of Gq protein α subunit and can inhibit the signal transduction of the Gq-coupled receptors including α_1_-adrenoceptor. Therefore, GCIP-27 not only ameliorated heart function but also increased food intake and body weight. Similarly, losartan can reduce afterload of the heart and improve heart function through blocking angiotensin II type 1 receptor, and subsequently decrease catecholamine and reduce body weight loss.

Additionally, it has been shown that GCIP-27 was superior at inhibiting ventricular remodeling compared with losartan [17]. In the present study, B-mode and M-mode echocardiography revealed that GCIP-27 was able to improve heart function. These results indicated that this polypeptide drug produced favorable effects on doxorubicin-induced CHF in rats. Although GCIP-27could lower blood pressure in spontaneously hypertensive rats, the hypotensive effect of GCIP-27 is significant weaker than that of losartan. And due to compensation, the normotensive animals, such as rats, dogs and patients, are less sensitive to hypotensive agents, such as losartan and nitroprusside, etc., than hypertensive subjects [32, 33]. We have also ever observed that GCIP-27 had no influence on the blood pressure in normotensive rats [17,19]. In doxorubicin-induced heart failure rats, blood pressure and hemodynamic parameters showed a slight decrease or no change [34, 35]. Therefore, the hemodynamics factors contributed not as much as remodeling to the mechanism for GCIP-27-induced improvement of heart function.

In the current study, chloral hydrate was used to anesthetize the rats before measuring cardiac function and tissue collection. Although chloral hydrate has various adverse effects [36,37], such as not providing analgesia, prolonging recovery time after surgery, inducing mutagenesis and carcinogenesis, it is considered by some a good sedative-hypnotics for animals. Experimental anesthesia based on an intraperitoneal injection of chloral hydrate is believed to have minimal effects on cardiovascular function or reflexes, while ketamine and pentobarbital sodium can lead to a significant decrease in heart function indexes and survival rate [36, 38]. Therefore, chloral hydrate is occasionally still used as an anesthetic agent in the laboratory [39–41]. Even though chloral hydrate has not been recommended for animal euthanasia in the CCAC guidelines, it was in line with the 10 general guiding principles listed in "*CCAC guidelines on*: *euthanasia of animals used in science*" (http://www.ccac.ca/Documents/Standards/Guidelines/Euthanasia.pdf), because it can result in rapid loss of consciousness and cause little distress and pain to the animals when used terminally. Meanwhile, gaseous anesthetics such as isoflurane may present health hazards to humans if not properly scavenged, for which special equipment is needed.

As the main contractile protein of myocardium, myoglobulin constitutes 60% of the mass of the entire heart and consists of one pair of MHCs and two pairs of MLCs. The two types of MHCs are α-MHC and β-MHC. The myocardial MHC phenotype changes correspond to the different stages of growth and development [42]. Accumulating research has suggested that the essence of myocardial remodeling is the process of the transformation of the myocardial cell phenotypes [43]. The readjustment of the myocardial contractile proteins occurs in all types of emergency situations, and the change from α-MHC to β-MHC is regarded as a molecular marker for hypertrophied or failing myocardium [44, 45]. In this research, we observed that GCIP-27 clearly increased the myocardial expression of α-MHC and decreased β-MHC expression in rats with CHF. GCIP-27 is able to correct the imbalance of myosin heavy chain expression and thereby enhance myocardial contractility.

Additionally, GCIP-27 treatment maintained the activity of the sarcoplasmic reticulum Ca^2+^ ATPase (SERCA) 2a and reversed the intracellular calcium overload as well. In cardiac muscle, the sarcoplasmic reticulum plays an important role in excitation-contraction coupling through the regulation of intracellular free-Ca^2+^ concentrations [46]. Muscle relaxation is initiated by Ca^2+^ transport from the cytosol into the sarcoplasmic reticulum by cardiac SERCA2a. The downregulation of SERCA2a has been reported to be a sign for the transition from compensated hypertrophy to a decompensated stage of CHF [47]. Many observations have suggested that the downregulation of SERCA2 might occur through a protein kinase C (PKC)-related process [48–50].

The PKC family consists of 15 isoenzymes, including PKCα, PKCβ, PKCε, PKCδ, PKCη and PKCμ. A study in a PKCε-knockout mouse model demonstrated that PKCε expression is not required for cardiac function under normal physiological conditions; however, PKCε activation is necessary for cardioprotection in myocardial hypertrophy and heart failure [51]. PKCα and PKCβ increase their expression and thereby decrease the contractile ability of cardiomyocytes during myocardial hypertrophy and heart failure [52–54]. It is reported that the postnatal cardiac-specific overexpression of the PKC-β isoform in transgenic mice caused cardiomyopathy with LV hypertrophy and *in vivo* cardiac dysfunction [55]. All of the Gq-coupled receptors associated with remodeling in the myocardium, including endothelin ET_1_ receptor, type I angiotensin II receptor, and the α_1_ adrenergic receptor, lead to the progression of myocardial remodeling through PKC. Gq overexpression in the mouse heart has been associated with PKC activation and dilated cardiomyopathy with overt heart failure [56]. In addition to PKC, several intracellular signaling pathways have been implicated to induce cardiac hypertrophy and CHF. The interconnectivity between PKC isoforms and the mitogen-activated protein kinase (MAPK) signaling cascade has been reported in many cell types [57, 58]. In particular, a number of studies have suggested that PKC and extracellular signal-regulated protein kinase 1/2 (ERK 1/2) might be concordantly regulated in the process of cardiac hypertrophy, extending to CHF [59]. It has been reported that the activation of ERK activity promotes a compensated form of hypertrophy [60, 61]. In this study, we observed that GCIP-27 could obviously increase PKCε expression in the rats with chronic heart failure, as well as reduce PKCβII expression. Simultaneously, ERK1/2 was activated by GCIP-27.

Dox induced heart failure through increasing oxidative stress, inflammation and apoptosis of cardiomyocytes [62]. In this process, Gαq-PKCε signaling is involved [55]. It has been reported that overexpression of Gαq resulted in obvious hypertrophic growth and apoptosis of cardiomyocytes and heart failure, and activating of PKCε was able to blunt apoptosis and therefore heart failure [63, 64]. As an imitation peptide of Gαq, GCIP-27 exerted anti-apoptosis effects by elevating expression of Bcl-2 and reducing that of Bax.

As a peptide, transport across the cell membrane is critical for GCIP-27 to produce its effects. In this systematic study, we found [16] that GCIP-27 could be transported through the plasmalemma in a time- and concentration-dependent manner, which was mediated by an energy-dependent endocytosis process. This peptide could preferentially enter myocardial cells and VSMCs, which is especially beneficial for the treatment of cardiac hypertrophy and CHF.

In conclusion, GCIP-27 could beneficially influence heart function and delay the onset of doxorubicin-induced CHF in rats. The regulation of the PKCβII and ε isoforms and ERK1/2 was involved in the intracellular signaling pathways leading to CHF. PKC–ERK1/2 signaling might represent the underlying mechanism responsible for the beneficial effect of GCIP-27.
